# A simultaneous [^11^C]raclopride positron emission tomography and functional magnetic resonance imaging investigation of striatal dopamine binding in autism

**DOI:** 10.1038/s41398-020-01170-0

**Published:** 2021-01-11

**Authors:** Nicole R. Zürcher, Erin C. Walsh, Rachel D. Phillips, Paul M. Cernasov, Chieh-En J. Tseng, Ayarah Dharanikota, Eric Smith, Zibo Li, Jessica L. Kinard, Joshua C. Bizzell, Rachel K. Greene, Daniel Dillon, Diego A. Pizzagalli, David Izquierdo-Garcia, Kinh Truong, David Lalush, Jacob M. Hooker, Gabriel S. Dichter

**Affiliations:** 1 Athinoula A. Martinos Center for Biomedical Imaging, Department of Radiology, Massachusetts General Hospital, Harvard Medical School, Charlestown, MA 02129 USA; 2 Department of Psychiatry, University of North Carolina-Chapel Hill, Chapel Hill, NC 27514 USA; 3 Department of Psychology and Neuroscience, University of North Carolina-Chapel Hill, Chapel Hill, NC 27514 USA; 4 Joint Department of Biomedical Engineering, University of North Carolina at Chapel Hill and North Carolina State University, Raleigh, NC USA; 5 UNC-Chapel Hill Department of Radiology and Biomedical Research Imaging Center (BRIC), Chapel Hill, NC 27514 USA; 6 Carolina Institute for Developmental Disabilities, University of North Carolina at Chapel Hill School of Medicine, Chapel Hill, Chapel Hill, NC 27510 USA; 7 Center for Depression, Anxiety and Stress Research, McLean Hospital, Belmont, MA USA; 8 Department of Biostatistics, University of North Carolina-Chapel Hill, Chapel Hill, NC 27514 USA

**Keywords:** Molecular neuroscience, Autism spectrum disorders

## Abstract

The social motivation hypothesis of autism posits that autism spectrum disorder (ASD) is characterized by impaired motivation to seek out social experience early in life that interferes with the development of social functioning. This framework suggests that impaired mesolimbic dopamine function underlies compromised responses to social rewards in ASD. Although this hypothesis is supported by functional magnetic resonance imaging (fMRI) studies, no molecular imaging study has evaluated striatal dopamine functioning in response to rewards in ASD. Here, we examined striatal functioning during monetary incentive processing in ASD and controls using simultaneous positron emission tomography (PET) and fMRI. Using a bolus + infusion protocol with the D2/D3 dopamine receptor antagonist [^11^C]raclopride, voxel-wise binding potential (BP_ND_) was compared between groups (controls = 12, ASD = 10) in the striatum. Striatal clusters showing significant between-group BP_ND_ differences were used as seeds in whole-brain fMRI general functional connectivity analyses. Relative to controls, the ASD group demonstrated decreased phasic dopamine release to incentives in the bilateral putamen and left caudate, as well as increased functional connectivity between a PET-derived right putamen seed and the precuneus and insula. Within the ASD group, decreased phasic dopamine release in the putamen was related to poorer theory-of-mind skills. Our findings that ASD is characterized by impaired striatal phasic dopamine release to incentives provide support for the social motivation hypothesis of autism. PET-fMRI may be a suitable tool to evaluate novel ASD therapeutics targeting the striatal dopamine system.

## Introduction

The social motivation hypothesis of autism proposes that functional disruptions in brain circuits supporting social motivation constitute a primary deficit that contributes to social communication impairments^1^. In particular, this framework posits that social communication symptoms in autism spectrum disorder (ASD) reflect decreased motivation to engage in reciprocal social behaviors throughout the development that results in fewer experiences with social rewards^2^. When children with ASD lack the motivation to participate in activities where social skills are typically forged, the resulting impoverished social environment compounds social impairments and negatively impacts the development of social communication^3^.

Social motivation is supported by the same substrates that govern other motivated behaviors, including ascending dopamine (DA) projections from the ventral tegmental area to the striatum and prefrontal cortex, forming a DA pathway sensitive to reward magnitude and probability^4^. This DA system mediates responses to social and nonsocial incentives^5^, and striatal DA transmission influences social behaviors^6^. Numerous functional magnetic resonance imaging (fMRI) studies have reported that ASD is characterized by decreased striatal responses to rewards^7,8^, highlighting striatal involvement in impaired social motivation in ASD^9^. In addition, impaired striatal functioning in ASD is implicated by altered effort-based decision-making for rewards^10^, polymorphisms of the DA D4 receptor gene, and the DA transporter gene are related to challenging behaviors^11^ and repetitive behaviors^12^ in ASD, and there are links between polymorphisms of the DA-3-receptor gene and striatal volumes and repetitive behaviors in ASD^13^. Furthermore, oxytocin abnormalities in ASD^14^, reports of the therapeutic effects of intranasal oxytocin administration for treating ASD symptoms^15^, and the effects of oxytocin on striatal responses to rewards in ASD^16^ support an etiologically relevant role for mesolimbic DA functioning in ASD: there are oxytocin projections within the mesolimbic DA system^17^, and oxytocin receptor activation plays an important role in the activation of reward pathways during prosocial behaviors^18^. Finally, the valproic acid model of ASD^19^ causes a cascade of neurobiological changes, including excitatory/inhibitory neural imbalances linked to increased basal DA in the frontal cortex^20^, hyperactive mesocortical DA in response to stress^21^, and changes in locomotor behavior akin to that observed in striatal DA-depleted animals^22^.

Despite converging evidence supporting the involvement of striatal DA impairments in the pathophysiology of ASD, no molecular imaging study has investigated striatal DA functioning in ASD. The goal of this study was to use simultaneous fMRI and positron emission tomography (PET) with the D2/D3 dopamine receptor antagonist [^11^C]raclopride to investigate striatal functioning during incentive processing in ASD. Neutral and rewarding incentives were presented during a behavioral fMRI task, and a bolus + infusion [^11^C]raclopride PET paradigm allowed measurement of both DA tone and phasic DA release in response to incentives. We hypothesized that the ASD group would be characterized by decreased striatal phasic DA release in response to incentives relative to a control group. We also hypothesized that, compared to controls, the ASD group would exhibit abnormal functional connectivity, assessed by fMRI, between striatal seed regions that showed reduced phasic DA release and their functional targets. Finally, exploratory analyses examined relations between striatal phasic DA release and symptom severity in the ASD group.

## Materials and methods

Procedures were in accordance with the ethical standards of the UNC-Chapel Hill (UNC) institutional research board and with the 1964 Helsinki declaration, and its later amendments or comparable ethical standards. The PET protocol was approved by the UNC Radioactive Drug Research Committee. Written informed consent was obtained prior to inclusion in the study. Participants provided informed consent and did not require surrogate consent.

### Participants

Participants with ASD and typically developing controls were recruited via the UNC Autism Research Registry and a university email listserv, respectively. Groups were matched on age, sex, and IQ. Exclusion criteria for both groups included lack of fluent phrase speech, IQ < 75, known sensory deficits, history of neurological injury, and MRI or PET contraindications. The control group had no lifetime psychiatric diagnoses, assessed by the Structured Clinical Interview for DSM-5 (SCID-5-RV)^23^. The ASD group had no current diagnosis of substance abuse or mood disorders, and no lifetime psychiatric diagnosis except for ASD, assessed by the SCID-5-RV.

Potential control participants completed the self-report version of the Social Communication Questionnaire (<15 cutoff) to rule out possible ASD symptoms^24^ and a screener for intellectual functioning (the North American Adult Reading Test (NAART)^25^. To aid group matching, control participants with NAART estimated IQ scores >120 were excluded. Eligible participants completed an in-person assessment that included the SCID-5-RV, the Wechsler Abbreviated Scale of Intelligence (WASI)^26^, the self-report Social Responsiveness Scale, Second Edition (SRS-2)^27^, a dimensional measure of ASD symptoms, and the “Reading the Mind in the Eyes” Test, Revised Version (RMITE)^28^, a measure of theory-of-mind. The ASD group also completed module 4 of the Autism Diagnostic Observation Schedule-2 (ADOS-2)^29^ administered by a reliable assessor (JLK or RKG) to confirm ASD diagnoses. Eligible participants were then scheduled for the PET-MR scan. Participants received $20/h for the assessment and $160–200 (based on task performance) for the scan.

Sample sizes were chosen so that the study would be powered to detect group differences with respect to a >15% change in DA binding with a power of 0.85 and a false discovery rate of 2.5% assuming a standard deviation in DA-mediated binding potential changes of 0.3. Twenty-six individuals with ASD and 34 controls (ages 19–29 years) provided written informed consent. Of these 60 potential participants, 23 were ineligible after in-person evaluation (11 controls, 12 with ASD) and one declined participation. Of the 36 participants who completed scanning, data from 22 were analyzable: 14 participants were not included due to problems with the PET injection or scanner (4), incomplete/missing data due to technical difficulties (8), abnormally low and noisy PET counts (1), and excessive motion during the PET scan (1). The final sample with analyzable PET data included 10 participants with ASD (all male; all white; 1 Hispanic) and 12 controls (10 males; 8 white, 2 Black or African American, 1 Asian, 1 race not reported; 2 Hispanic).

### Simultaneous PET-MR scanning

**Fig. 1:**
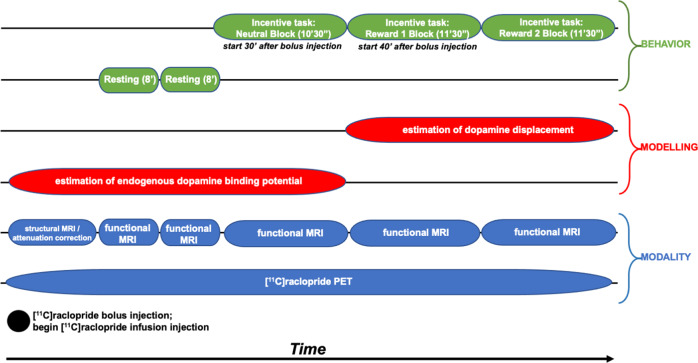
Timing of data collection, data modeling, and participant behavior during scanning. Three task blocks were presented during which fMRI data were collected simultaneously to the PET acquisition.

Participants completed a simultaneous PET-MR scan on a Siemens Biograph mMR scanner at the UNC Biomedical Research Imaging Center using a bolus + infusion protocol with a planned *K*_bol_ of 105 min^30^. PET acquisition took place for 63 min. Approximately 1 min after the scan began, a bolus injection of [^11^C]raclopride was administered after which the infusion injection of [^11^C]raclopride was administered using a Medrad® Spectris Solaris® EP MR Injection System (radioactivity was limited to 15 mCi over the bolus and infusion and mass dose did not exceed 10 µg (with a specific radioactivity at the bolus time of injection >0.4 Ci/µmol)). A 6 min structural T1-based MR sequence was obtained (FOV = 256 mm, 1 × 1 × mm resolution, TR = 2530 ms, TE = 1.69 ms, flip angle = 7 degrees) for anatomical localization, spatial normalization of imaging data, and generation of attenuation correction maps^31^, in addition to localizer and attenuation correction scans. Then, two resting-state scans were obtained (echo planar imaging, FOV = 212 mm, 3.312 × 3.312 × 3.3 mm resolution, TR = 3000, TE = 30 ms, flip angle = 90 degrees). Next, three task blocks were presented during which fMRI data were collected simultaneously to the PET acquisition. See Fig. 1 for timing of data collection, data modeling, and participant behavior.

### Behavioral task during PET and fMRI scanning

Participants completed a monetary incentive delay task^32^ modified for use in PET-MR studies (by DD and DAP at McLean Hospital). The task presented a 10′30″ neutral block (no monetary rewards delivered) followed by two 11′30″ reward blocks (monetary rewards of varying magnitudes delivered). The task was programmed such that ~75% of each participant’s responses were successful based on individualized reaction time (RT) performance.

Participants first completed two 8′09″ resting-state fMRI runs with eyes open to allow for tracer uptake. Next, participants completed a monetary incentive delay task^32^ modified for use in PET-MR studies. The task was presented using PsychoPy software version 1.84.1^33^. The task presented a 10′30″ neutral block followed by two 11′30″ reward blocks. As shown in Supplemental Materials I, on each trial (6.37–15.17 s), participants saw a blue polygon cue (1.5 s), followed by a green circle target (0.367 s) and an outcome (1.5 s); these stimuli were separated by jittered interstimulus and intertrial intervals during which a fixation cross was shown. The task required making a speeded button press with the right index finger upon seeing the target. During the neutral block, participants completed 63 trials that started with a square cue. No monetary rewards were delivered on these trials. Instead, sufficiently speeded button presses resulted in the presentation of a gray rectangle as a “no-reward” outcome. The other outcomes indicated either no response (“No Response!”), the response was too quick (within 100 ms of the target presentation: “Too Fast!”), or it was made after an adaptive RT threshold (“Too Slow!”) that was programmed such that ~75% of each participant’s responses were successful.

The neutral block was followed by four reward runs, combined into two blocks (number of trials per reward run: block 1: 34/33, block 2: 33/34). The neutral and reward blocks were separated by a brief break. In the reward blocks, different polygon cues (square, triangle, pentagon, and hexagon) indicated that trials could result in no-reward (gray rectangle) or a small (50 cents), medium (1 dollar), or large reward (5 dollars), respectively; the assignment of the four polygons to the four outcomes was stable across the reward blocks and counterbalanced across participants. Successful trials (i.e., trials with sufficiently speeded button presses) ended with images depicting the no-reward, small, medium, and large reward outcomes. Unsuccessful trials yielded the same feedback as in the neutral block (“Too Fast!”, “Too Slow!”, or “No Response!”). Following each neutral and reward block, participants rated cues and outcomes using a nine-point Likert scale with anchors of “very negative” and “very positive” at the ends and “neutral” in the center.

This MID task includes novel features designed to maximize detection of DA release in the PET-MR environment. First, the first reward block begins ~40 min after the [^11^C]raclopride bolus injection after the target/reference ratio stabilized; the long uptake period serves as a baseline scan (i.e., baseline binding potential is estimated from injection up to the start of the reward block). Second, ~75% of reward trials result in reward feedback, including many $5 rewards; this success rate is higher and the large rewards are larger than in many MID studies to enhance incentive motivation, which should be evident in stronger signals related to reward anticipation and consummation^34^. Third, while most MID versions use explicit reward and neutral cues that make the potential outcome of each trial clear, the current design forces participants to learn which cues predict which reward magnitudes by experience. By adding associative learning, the current design should enhance sensitivity to positive prediction errors (and other learning-related signals) encoded by phasic DA release^35^.

### PET analysis

Post-scan reconstruction of the PET data used 1 min frames^36^. BP_ND_ was defined as the ratio of selectively bound ligand to nondisplaceable ligand in the tissue at equilibrium using the two-part simplified reference tissue model (SRTM). In order to identify between-group differences in striatal BP_ND_, a *Z*-score statistical map representing the contrast of ASD > control was created by comparing group-level voxel-wise BP_ND_ (reward > neutral) maps and thresholded at  Z > 2.3 (i.e., *p* < 0.012). This map was subsequently masked by the bilateral caudate nucleus, putamen, and nucleus accumbens regions from the Harvard–Oxford probabilistic atlas (thresholded at 25% and binarized). For each significant cluster, condition-specific BP_ND_ values were extracted from each participant and analyzed using group (ASD, control) × condition (reward, neutral) ANOVAs. Reduced BP_ND_ is interpreted to mean an increase in endogenous DA (i.e., competition with [^11^C]raclopride). Results for the contrast of ASD > control, reward > neutral, signify increased BP_ND_ or decreased phasic DA release to the reward condition, relative to the neutral condition, in the ASD group compared to controls. For a complete description of PET analyses see Supplemental Materials II and Sander and colleagues^36^.

### fMRI general functional connectivity analysis

We used general functional connectivity (GFC) to examine whole-brain connectivity with striatal seed regions, in which we observed significant differences in BP_ND_ between diagnostic groups. GFC, a method that combines resting-state and task fMRI data, offers better test-retest reliability and higher estimates of heritability than intrinsic connectivity estimates from the same amount of resting-state data alone^37^. In the present study, where combining the two resting-state runs and three task blocks yields 49′30″ of fMRI data for connectivity analyses, GFC also offers the advantage of longer durations of fMRI data to be analyzed. This is critical given that >25 min of fMRI data are needed to reliably detect individual differences in connectivity^38–40^.

Voxel-wise whole-brain connectivity was evaluated using the CONN Toolbox’s seed-to-voxel analysis. Functional images were preprocessed with the default preprocessing pipeline in the SPM12 CONN functional connectivity toolbox, version 19c (ref. ^41^). Steps included: resampling to 2 × 2 × 2-mm voxels and unwarping, centering, slice time correction, normalization to MNI template, outlier detection (ART-based scrubbing), and smoothing to an 8 mm Gaussian kernel. Motion parameters were entered as multiple regressors and images with framewise displacement >0.5 mm or global BOLD signal changes >3 SD were flagged as potential outliers and regressed out^42^. For most of the sample (*n* = 19) all five runs of functional data were analyzable and all participants had at least three analyzable runs. Reasons for excluded runs were: technical errors (2), striation artifacts (1), and excessive motion (2). There were no significant differences between groups on average motion, *t*(20) = 0.35, *p* > .05 (two-sided), or average global BOLD signal changes, *t*(20) = 1.07, *p* > .05 (two-sided).

### Exploratory fMRI activation analysis

The main objectives of this study were to investigate striatal DA release in ASD and GFC with striatal regions showing impaired DA release in ASD. Therefore, fMRI activation analyses and task-based generalized psychophysiological interactions (gPPI) analyses are presented as supplementary.

The first four volumes of each functional run were discarded to allow for steady state equilibrium. Functional data were preprocessed using FSL FEAT version 6.0 (Oxford Centre for Functional Magnetic Resonance Imaging of the Brain (FMRIB), Oxford University, U.K.). Preprocessing was applied as follows: brain extraction for non-brain removal^43^, motion correction using MCFLIRT^44^, and spatial smoothing using a Gaussian kernel of FWHM 6 mm and high-pass filtering. Functional images were co-registered to structural images in native space, and structural images were normalized into a standard stereotaxic space (Montreal Neurological Institute). Registrations used an intermodal registration tool^43,45^. Voxel-wise temporal autocorrelation was estimated and corrected using FMRIB’s Improved Linear Model^46^. Models included nuisance covariates of 24 realignment parameters (six motion parameters plus their six temporal derivatives, and quadratic terms of these 12 regressors). Volumes with framewise displacement >0.9 mm, identified via fsl_motion_outliers, were censored^47^. Runs with >20% of volumes censored were discarded. One participant in the ASD group was discarded based on excessive motion.

To examine fMRI responses during reward anticipation, the contrast between neutral and reward trials of all magnitudes (small, medium, and large) from the onset of the cue to the end of the fixation period (i.e., during the cue and the target) was examined. To examine fMRI responses during reward outcomes, the contrast between successful and unsuccessful outcomes (i.e., successful vs. unsuccessful reward outcomes on reward trials of all magnitudes (small, medium, and large)) was examined. Group-wise activation images were calculated by a mixed effects higher-level analysis using Bayesian estimation techniques with FMRIB Local Analysis of Mixed Effects (FLAME 1 + 2)^48^, with outlier de-weighting and sex was included as a covariate.

Because the sample size of the current study is smaller than other fMRI studies of reward processing in ASD^7^, and because our a priori hypotheses were focused on the striatum, a structure that is comprised of anatomically small regions that typically do not survive stringent correction, we applied a small volume correction for the striatum, with a voxel-wise threshold of *Z* > 2.3 (*p* < 0.012) and minimum cluster size of 20, as has been done in prior fMRI studies examining the striatum^49–51^.

### Exploratory gPPI analysis

Preprocessing steps were nearly identical to those described for GFC analysis, with the exception of including high-pass filtering <0.008 Hz (vs. band-pass filtering <0.008 Hz or >0.09 Hz). Regarding motion exclusion: one control participant had a task run excluded due to technical errors; one control participant had a task run excluded due to striation artifacts; and one ASD participant had two task runs excluded due to excessive motion.

Using CONN Toolbox, voxel-wise models evaluated whole-brain connectivity with the five striatal seeds that demonstrated ASD > control group differences for the contrast of (reward > neutral) BP_ND_ values, reflecting greater difference in phasic DA release in the reward relative to the neutral condition in the control group relative to the ASD group. For each participant, mean fMRI time courses (i.e., physiological regressors) were extracted from seed regions for each task run, then multiplied by each psychological regressor of interest (i.e., task condition: reward and non-reward) to form the PPI interaction terms. The gPPI model included physiological and psychological regressors, as well as their interaction terms to describe the unique effect of these interactions above and beyond the main effects of seed time courses and task conditions. The primary contrasts of interest are match those described in the fMRI activation analytic plan: reward anticipation is defined as the contrast between reward vs. neutral trials from the onset of the cue to the end of the fixation period and reward outcome is defined as the contrast between successful vs. unsuccessful reward outcomes on reward trials of all magnitudes.

## Results

All statistical analyses report two-sided significance tests.

### Study participants

Groups did not differ in sex, race, or ethnicity distributions, Fisher’s exact test *p*’s > 0.34. As depicted in Table 1, the ASD group differed from controls with respect to scores on the SRS-2 and RMITE, but not IQ, SES, or age. [^11^C]raclopride dose did not differ between groups; for the ASD and control group, the mean (SD) dose was 12.39 (0.98) and 11.73 (2.14) mCi, respectively, *W* = 52, *p* = 0.63.

**Table 1:** Participant characteristics.

	ASD (*n* = 10)	Control (*n* = 12)	Test statistic	*p* Value
	*M* (SD)	*M* (SD)		
ADOS-2 CSS	8.3 (2.1)	—	—	—
SRS-2 total *T* score	67.6 (12.2)	46.67 (7.2)	*t* (20) = 5.01	<0.001**
WASI-2 full-scale IQ	116.5 (11.9)	120.67 (8.9)	*t* (20) = 0.94	0.36
RMITE	22.6 (3.50)	27 (2.30)	*t* (20) = −3.54	0.002*
SES	38.3 (14.4)	39.67 (11.5)	*t* (20) = 0.25	0.81
Age	24.9 (3.6)	25.67 (4.3)	*t* (20) = 0.45	0.66
Sex	10 ♂	10 ♂, 2♀	*χ*^2^ = 0.22	0.48

*ASD* Autism spectrum disorder group, *Control* control group, *M* mean, *SD* standard deviation, *ADOS-2 CSS* Autism Diagnostic Observation Schedule, 2nd edition Calibrated Severity Score^84^, *SRS-2* Social Responsiveness Scale, Second Edition, *WASI-2* Wechsler Abbreviated Scale of Intelligence, 2nd edition^26^, *RMITE* Reading the Mind in the Eyes Test, Revised Version^28^, *SES* socioeconomic status measured by the Hollingshead Four Factor Index of Socioeconomic Status^85^.

*p < 0.005; **p < 0.0001.

### PET results

**Fig. 2:**
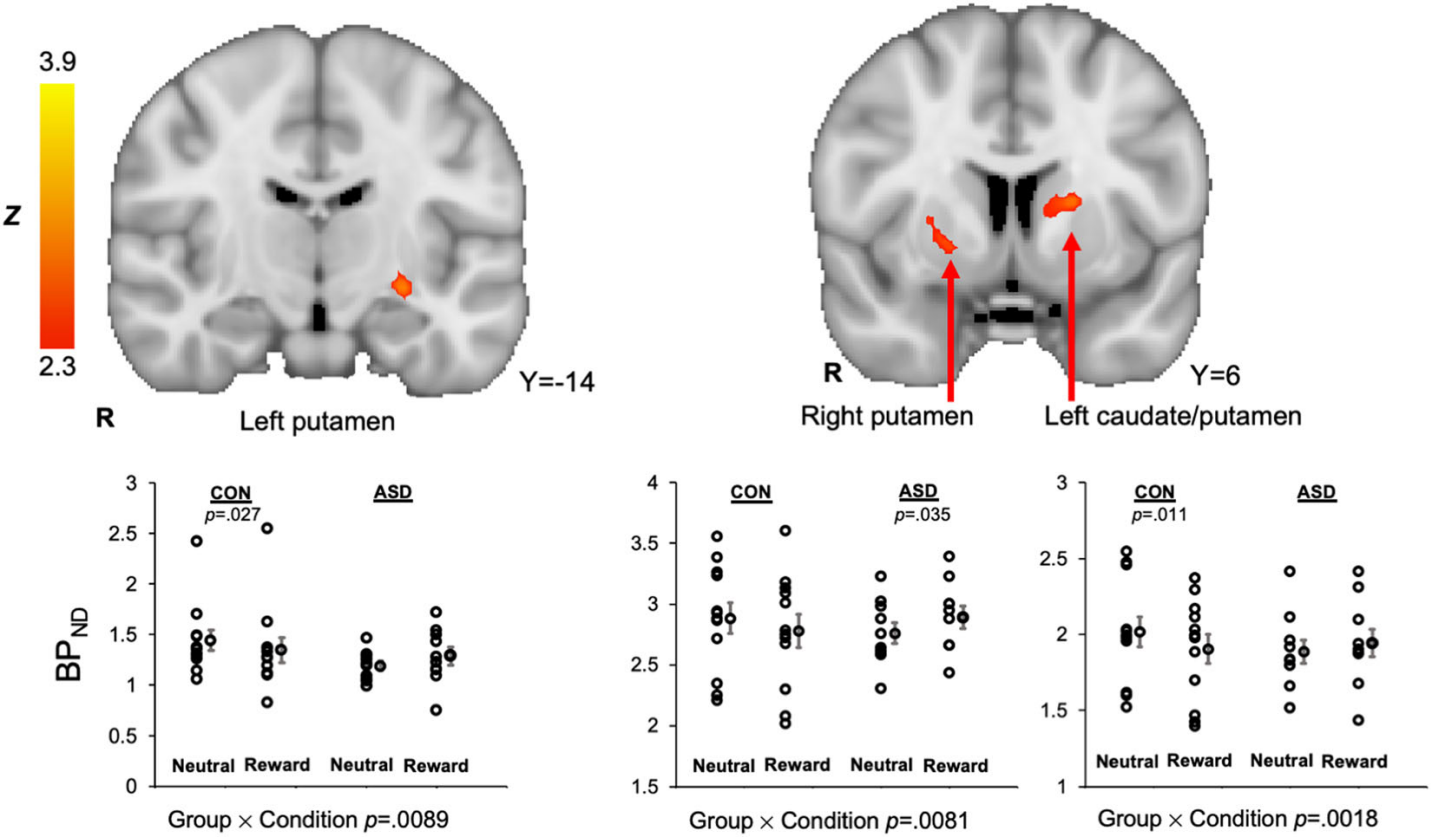
Striatal clusters that showed ASD > control [^11^C]raclopride BP_ND_ (reward > neutral), signifying decreased phasic release of dopamine to rewards in the ASD group relative to the control group, were evident in the left putamen, right putamen, and a cluster that spanned the left caudate nucleus and putamen. For all clusters, the group (ASD, control) × condition (reward, neutral) interaction effect on [^11^C]raclopride BP_ND_ values were significant. For each dot plot, individual data points are displayed next to the group average with standard error bars.

Three striatal clusters showed ASD > control group differences for the contrast of (reward > neutral) BP_ND_ values, reflecting greater difference in phasic DA release in the reward relative to the neutral condition in the ASD group relative to control group. Clusters were located in the right putamen, left putamen, and left caudate nucleus/left putamen. Condition-specific BP_ND_ values were extracted for each participant. In all conditions, there was a significant group × condition interaction, *F*’s(1.20) > 8.6, *p*’s < 0.009 (Levene’s tests did not indicate unequal variances, *p*’s > 0.05; see Fig. 2 and Table 2). In general, the ASD control group exhibited increased BP_ND_ in the reward condition relative to the neutral condition, interpreted as decreased phasic DA release to rewards, whereas the control group showed the inverse response. Because the left caudate nucleus/left putamen cluster contained white matter voxels, this region was further analyzed, and subregional analysis confirmed the presence of the effect in the gray matter of left caudate and putamen (described in Supplemental Materials III). Furthermore, region-of-interest analyses using the 1 mm striatum structural atlas^52^ showed a significant difference in the right putamen and trending differences in the left putamen and left caudate (presented in Supplementary Materials IV), supporting the aforementioned SRTM results. Finally, one cluster in the right caudate nucleus demonstrated the opposite pattern of group differences for the contrast of (reward > neutral) BP_ND_ values: relative to controls, the ASD group exhibited decreased BP_ND_ (or increased DA) in the reward relative to the neutral condition, group × condition interaction, *F*(1,20) = 7.5, *p* < 0.05. Power and effect size analyses for the central PET findings are presented in Supplementary Materials V.

**Table 2:** Striatal clusters demonstrated ASD > control group differences for the contrast of (reward > neutral) BP_ND_ values at the threshold of *z* > 2.3, reflecting greater difference in phasic DA release in the reward relative to the neutral condition in the control group relative to the ASD group.

Cluster Label	Cluster Size (voxels)	Max *Z* value	Max *X*	Max *Y*	Max *Z*
Left caudate nucleus / putamen	87	3.95	−18	2	10
Right putamen	41	2.84	22	6	−2
Left putamen	40	3.22	−26	−16	−8

### fMRI general functional connectivity results

**Fig. 3:**
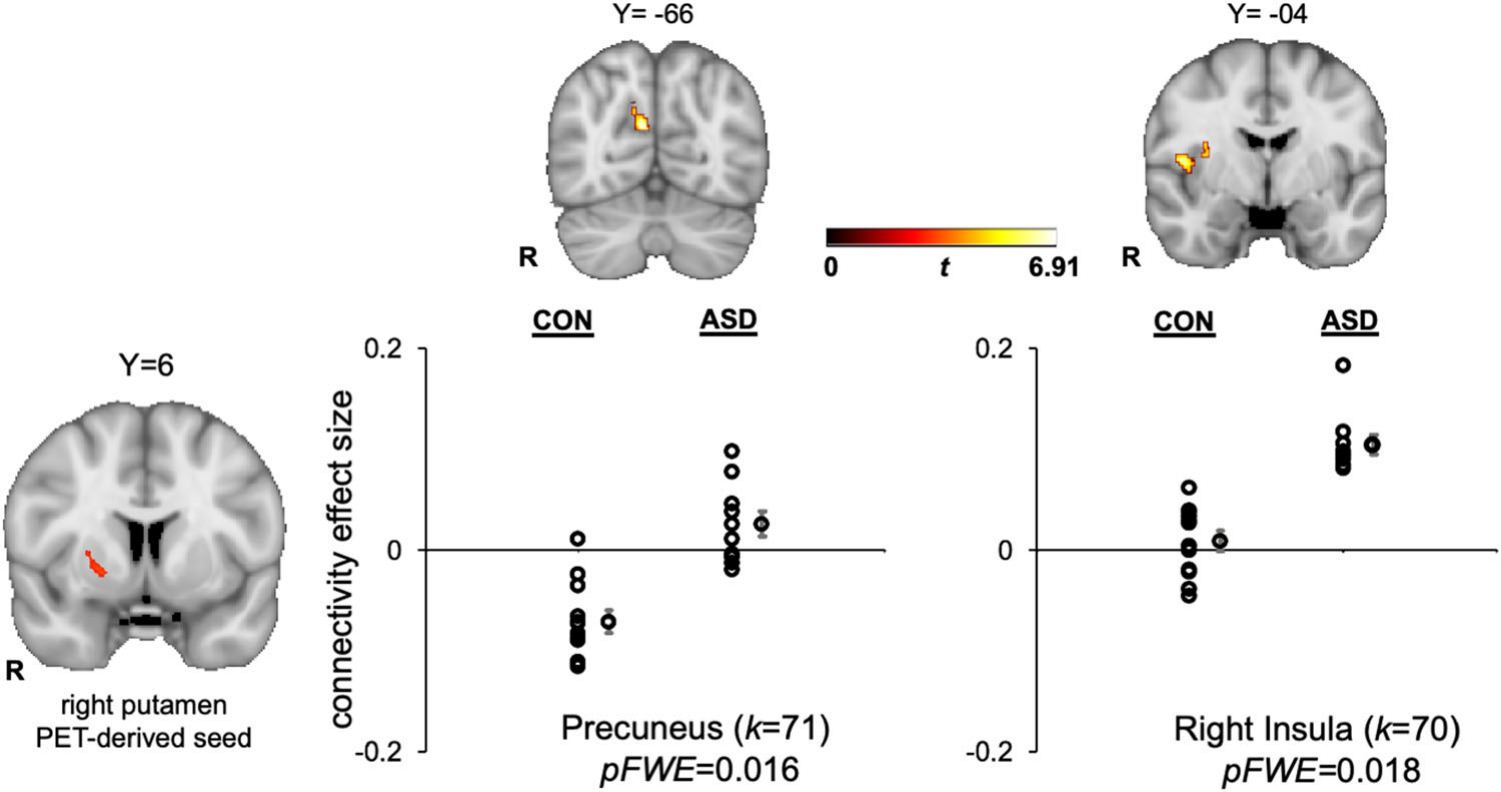
ASD > control differences in general functional connectivity between the PET-derived right putamen seed and precuneus (MNI coordinates: *X* = 8, *Y* = −66, *Z* = 24) and right insula (MNI coordinates: *X* = 48, *Y* = −4, *Z* = 10). Clusters are displayed in MNI152 space at peak coordinates for each target region and family-wise-error cluster corrected at *p* < 0.05. The dot plots show the effect size for each target region, represented by the Fisher-transformed correlation coefficients, separated by group. Individual data points are displayed next to the group average with standard error bars. *k* = cluster size in voxels.

Whole-brain GFC analysis revealed significant group differences in connectivity with the PET-derived right putamen seed (based on group differences for the contrast of (reward > neutral) BP_ND_ values), but no other PET-derived striatal seeds. Compared to the control group, the ASD group exhibited relatively greater connectivity between the right putamen seed and the precuneus and right insular cortex (see Fig. 3).

### Exploratory fMRI activation results

Localizations were based on Harvard–Oxford cortical and subcortical structural probabilistic atlases. Cluster-corrected results during reward anticipation yielded no significant clusters that differentiated groups at a threshold of *z* > 2.3. Uncorrected results with a voxel-wise threshold of *z* > 2.3 (*p* < 0.012), and minimum cluster size of 20 shows that the ASD group demonstrated decreased activation in a 34-voxel cluster in the left putamen, as well as other cortical and cerebellar regions (see Supplemental Materials VI). The size of this left putamen cluster exceeded our planned small volume correction for the striatum based on approaches used in prior studies examining this region^49–51^. For completeness, other clusters showing ASD < control activation differences at this threshold and size are also included in Table 1, but findings outside of the striatum should be considered exploratory given that these are uncorrected results at a liberal threshold. Cluster-corrected results thresholded at *z* > 2.58 (*p* < 0.005) during reward outcomes indicate that the ASD group showed decreased activation in a large cluster in the anterior cingulate gyrus (1486 voxels) during reward outcomes.

### Exploratory generalized psychophysiological interactions results

Voxel-wise whole-brain gPPI was analyzed using CONN’s seed-to-voxel tool. To examine the reward anticipation period, the contrast between neutral and reward trials from the onset of the cue to the end of the fixation period (i.e., during the cue and the target) was tested. To examine the reward outcomes period, the contrast between successful and unsuccessful outcomes (i.e., successful reward outcomes vs. unsuccessful reward outcomes on reward trials of all magnitudes) was tested. During reward anticipation, there was a significant group difference in connectivity between the left putamen cluster that demonstrated group differences for the contrast of (reward > neutral) BP_ND_ values and a target region in the left orbital frontal cortex (see Supplementary Materials VII). There were no group differences in connectivity with any seed during reward outcomes.

### Task reaction time and valence ratings

Supplementary Materials VIII presents the results of analyses of task RTs and valence ratings to cues and outcomes.

### Correlations between striatal dopamine binding and ASD symptom severity

Exploratory correlational analyses between striatal dopamine binding and ASD symptom severity are presented in Supplemental Materials IX.

## Discussion

The social motivation hypothesis of autism proposes that impaired reward circuitry responses to social information give rise to social communication symptoms in ASD. Although numerous fMRI, electrophysiological, and behavioral studies have investigated this framework^53^, no previous molecular imaging study has directly investigated striatal DA functioning in ASD. In this study, we evaluated striatal DA functioning in ASD via simultaneous PET and fMRI during incentive processing using the D2/D3 dopamine receptor antagonist [^11^C]raclopride and a novel MID task. We supplemented our PET analysis by using fMRI to examine functional connectivity of striatal regions that showed impaired DA release in ASD.

Analysis of [^11^C]raclopride PET data revealed relatively decreased phasic DA release to rewards in the ASD group in several striatal clusters, including the putamen and caudate nucleus. The putamen and the caudate nucleus comprise the dorsal striatum, a structure centrally involved in reinforcement learning and goal-directed behaviors that are facilitated by dorsal striatal DA release^54,55^. Specifically, the dorsal striatum plays an important role in learning stimulus-action-outcome associations and stimulus-action coding^56^. Though the present study did not investigate reward learning, this pattern of impaired dorsal striatal DA release in the ASD group is consistent with the well-documented deficits in learning^57,58^ and flexible responses to environmental contingencies^59^ in ASD that may result from atypical computation of prediction errors^60,61^. The neuroimaging literature addressing reward learning in ASD has found decreased frontostriatal activity during both implicit and explicit social reward learning tasks^8,62,63^, underscoring the potential relevance of impaired reward learning to core ASD symptoms.

fMRI functional connectivity with PET-derived striatal seed regions was evaluated with a GFC approach. The only PET-derived seed region that showed GFC group differences was the right putamen. Greater connectivity was observed in the ASD group between the right putamen and the precuneus and right insular cortex. The precuneus has direct connections to the basal ganglia^64^, is involved in self-referential processing^65^ and has been linked to mentalizing deficits in ASD^66^. Notably, increased striatal connectivity with the precuneus during reward processing has been associated with depressive symptom severity in anhedonic patients with major depressive disorder (MDD)^67^. Thus, in the present context, increased connectivity between a putamen cluster demonstrating decreased phasic DA release and the precuneus may reflect a possible shared feature of ASD and MDD, though this interpretation is speculative pending follow-up studies designed to evaluate linkages between striatal DA functioning and depressive symptoms in ASD.

The right putamen PET-derived seed also demonstrated increased connectivity with the right insular cortex in the ASD group. The insular cortex, and in particular its anterior portion, is a critical hub for regulating large-scale brain network dynamics^68^ and is part of the salience network that integrates sensory, autonomic, and hedonic input to guide behavior^69^. In ASD, there is evidence that the insula plays a key role in social and nonsocial impairments^70^. A meta-analysis of functional neuroimaging ASD studies found insula hypoactivation in ASD during a range of social processing tasks^71^, suggesting that insula dysfunction may be central to the disorder^72^ given the multiple functions subserved by the insula, including attention and affective processing of salient social information^73^. Increased functional connectivity between the putamen and insula has been reported during rest in children with ASD, though this pattern of abnormal insular connectivity was not specific to the putamen, but rather observed in a number of dorsal and ventral striatal seeds and cortical regions^74^. The finding in the present context of increased connectivity between a putamen cluster demonstrating decreased phasic DA release to rewards, and the right insular cortex highlights that striatal DA signals may drive the impaired functioning of various associative and limbic cortices implicated in the pathophysiology of ASD, though this interpretation is speculative pending follow-up studies designed to evaluate linkages between striatal DA functioning and insular connectivity in ASD.

Exploratory fMRI activation in response to the incentive task revealed decreased activation in the left putamen during reward anticipation and in the anterior cingulate gyrus during reward outcomes in the ASD group, though the former finding was at an uncorrected threshold. Broadly, these results are consistent with the literature documenting decreased neural responses to monetary rewards in ASD using fMRI^7^. Exploratory gPPI connectivity analyses revealed ASD > control group differences during reward anticipation in connectivity between the PET-derived left putamen cluster that demonstrated group differences for the contrast of (reward > neutral) BP_ND_ values and the orbital frontal cortex, a region implicated in oxytocin response in ASD^16^. Finally, exploratory correlational analyses in the ASD group revealed that decreased phasic DA release to incentives in the left and right putamen was related to worse theory-of-mind, a core ASD impairment^75^, highlighting the clinical significance of findings.

This study had several limitations. First, the sample size is small, though it is noteworthy that for each of the central PET findings representing group (control, ASD) × condition (reward, neutral) differences in BP_ND_ values in three striatal clusters (left putamen, right putamen, and left caudate/putamen, see Fig. 2), the effect size is at least 2.90 and power is at least 0.79 (see Supplemental Materials V). Second, the imaging task presented monetary rewards rather than social rewards. This design was to ensure a robust striatal DA response in the control group given the extant literature demonstrating striatal DA release to monetary rewards in nonclinical samples using [^11^C]raclopride^76,77^. Although the social motivation hypothesis of autism highlights impaired responses to social rewards in ASD, several studies report striatal dysfunction to both social and nonsocial rewards in ASD^7,53,78,79^. Thus, the present study has mechanistic relevance to address impaired social motivation responses in ASD. This study was also restricted to participants with higher cognitive abilities and may not represent the broader ASD population. In this regard, we have established PET-MR protocols for adults with ASD with lower intellectual functioning^80^ and recently completed a PET-MR study that included individuals with ASD with full-scale IQs ranging from 47 to 112^81^. Finally, two results should be interpreted with caution until replicated: (1) a cluster in the right caudate nucleus showed increased phasic DA release to rewards in the ASD group, a finding that was unexpected and in the opposite direction of other striatal PET clusters; and (2) the relations between phasic DA release in the left and right putamen, and performance on the theory-of-mind measure were only significant at an uncorrected threshold. It is also possible that unsuccessful trials during the scanner task elicited frustration. Finally, it is not possible with the current paradigm to discern whether group differences in striatal BP_ND_ reflect differences in phasic DA release vs. decreased binding affinity due to receptor trafficking (i.e., reduced binding affinity due to receptor desensitization or internalization)^36^.

In spite of these limitations, this study is the first PET-MR investigation of striatal DA functioning in ASD. Using [^11^C]raclopride in conjunction with a reward processing task, we report evidence consistent with impaired phasic DA release to rewards in the striatum in ASD. We further demonstrated that functional connectivity in the ASD group was increased between a PET-derived right putamen seed (that exhibited decreased phasic DA release to rewards) and the precuneus and right insula, suggesting a molecular mechanism that may address, in part, the pathogenisis of impaired functional brain networks in ASD. These results indicate that ASD is characterized by impaired striatal DA functioning, consistent with the social motivation hypothesis of autism, and highlights that PET-MR may be a suitable tool to evaluate novel treatments aimed at improving striatal DA functioning in ASD. More broadly, the use of simultaneous PET-MR represents an important means to address the heterogeneity of ASD^82^ by identifying individuals characterized by homogenous molecular etiologies. It additionally holds the promise of validating the molecular underpinnings of fMRI signals. Finally, it provides perhaps the most direct linkages possible between human disorders and preclinical animal models characterized by common molecular pathophysiologies^83^.

## Supplementary information

Supplementary Materials
