# Supplementary material for: A simultaneous [11C]raclopride positron emission tomography and functional magnetic resonance imaging investigation of striatal dopamine binding in autism
Source: Transl Psychiatry. 2021 Jan 11;11:33. doi: 10.1038/s41398-020-01170-0 (PMC7801430; doi:10.1038/s41398-020-01170-0)
Supplement: Supplementary file 1 — Supplementary Materials [file 41398_2020_1170_MOESM1_ESM.docx]

**Supplementary Materials**

**Supplemental Materials I:** The PET-MR monetary incentive task


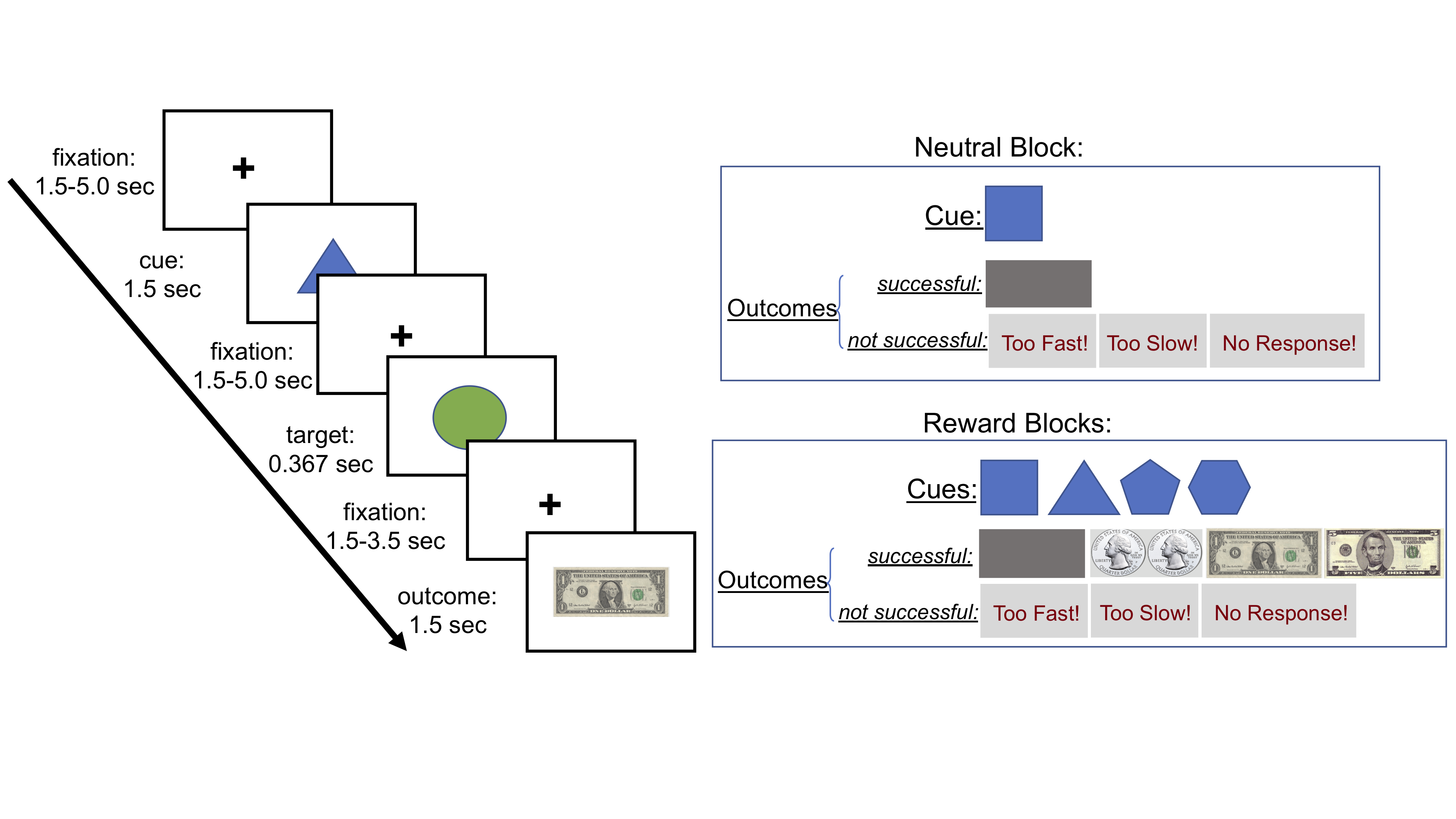


Each trial consisted of a cue phase and an outcome phase. Trials were presented first in a neutral block that consisted of only neutral trials and then in two reward blocks that consisted of neutral trials and reward trials of varying magnitudes (small, medium, or large).

**Supplemental Materials II:** PET analyses

A. Reconstruction

Attenuation maps including bone and sinus detail were created using the PseudoCT method ^1^, which uses the subject’s Dixon attenuation map and the T1 MPRAGE image to estimate a CT-equivalent attenuation map. The PseudoCT method showed high accuracy in a head-to-head comparison of several methods for MR-based attenuation correction ^2^.

PET images were reconstructed from list-mode data for 70 1-minute time frames starting at injection time using vendor-provided software (E7Tools, Siemens Healthineers). Reconstructions used the OS-EM algorithm for 3 iterations and 24 subsets and included corrections for attenuation, scatter, and randoms. The reconstruction grid was 344$\times$344 with 127 axial slices and a voxel size of 2.086mm $\times$ 2.086mm $\times$ 2.032mm.

B. Motion Correction

The dynamic PET images were corrected for motion using the Realign procedure of SPM12 ^3^. This method computes a rigid transformation for each time frame to align all to a common reference. The result of this procedure is a set of 70 $\times$ 1-minute dynamic images that are well aligned.

As a quality-control measure, the motion-corrected frames were observed in cine mode to detect possible errors. In all but two cases, the motion correction was found to achieve good alignment. For one subject we found a significant sudden movement over three frames that was not well corrected. Because this only affected three frames in the early portion of the scan, we chose to exclude those frames from the dynamic fits for that subject alone. In another subject, movement during the scan was found to be so substantial and uncorrectable that the subject was excluded from further analysis.

C. Subject-to-MNI Space Transformation

For each subject, a mapping from subject space to reference MNI space was determined as a composition of two transforms: first, a rigid transform to align the motion-corrected PET to the subject’s T1 MPRAGE image, and, second, a deformable transform to align the subject’s T1 MPRAGE image with the MNI reference T1 image. The rigid transform was found to be necessary in many subjects either due to (1) movement between the times that PET and T1 images were taken and/or (2) the motion correction procedure displacing the PET from the T1 by a few millimeters. The rigid transforms were determined manually by displaying the motion-corrected PET (20-minutes post-injection) overlaid on the T1 MRI in Slicer ^4^.

The deformable component was determined using the Dartel tools in SPM12 ^5^. This procedure solved for a mean intermediate reference among all subjects and the MNI reference based on segmentations of gray and white matter from the T1 MPRAGE images. Then, deformations for the individual subjects mapping to the MNI space were composed from subject->intermediate and inverse (intermediate->MNI) deformations. The result of this procedure was a set of rigid (PET->T1) and deformable (T1->MNI) transforms for each subject.

D. Subject-specific Atlases

With the individual transformations available, atlases defined in the MNI space were mapped to individual subject PET spaces by inverting the transforms determined in part C above. The AAL3 atlas ^5^, which is defined in 2mm MNI space, was transformed to each subject’s PET space and resampled to the 344$\times$344$\times$127 PET grid so that the atlas could be applied to each of the 70 motion-corrected PET images without further transformation. The AAL3 atlas was mapped using nearest-neighbor interpolation to preserve the integer labeling of its 170 regions.

In addition, the three-region 1mm striatum structural atlas ^6^, also defined in MNI space, was divided by left and right hemispheres (making six total regions), then transformed in the same way; however, this atlas was mapped using linear interpolation in order to weight edge voxels for partial volume effects.

E. BP_ND_ Estimates from Regional TACs

For each subject, the subject-specific 170-region AAL3 atlas was applied to each of the 70 motion-corrected PET images to obtain the mean PET intensity (in Bq/ml) in each region for each time frame. Thus, a time-activity curve (TAC) was obtained for each region.

The simplified reference tissue (SRTM) model ^7^ was applied to TAC data as follows. The SRTM model was applied with a two-part BP_ND_ component to account for the neutral (from injection time to start of reward task) and reward states:

$$h_{neutral}\left( t \right)= exp\left( \frac{-k_{2}t}{1+{BP}_{ND_{Neutral}}} \right)$$

$$h_{reward}\left( t \right)= = exp\left( \frac{-k_{2}t}{1+{BP}_{ND_{Reward}}} \right)$$

$$Q\left( t \right)= \sum_{t^{'}=0}^{t_{reward}-1} {TAC}_{ref}\left( t^{'} \right)h_{neutral}\left( t-t^{'} \right)+ \sum_{t^{'}=t_{reward}}^{end} {TAC}_{ref}\left( t^{'} \right)h_{reward}\left( t-t^{'} \right)$$

$$TAC\left( t;R_{1},k_{2}, {BP}_{ND_{Neutral}} , {BP}_{ND_{Reward}} \right)= R_{1}{TAC}_{ref}\left( t \right)+\left( k_{2}-\frac{R_{1}k_{2}}{\left( 1+{BP}_{ND_{Neutral}} \right)} \right)Q\left( t \right)if 0<t<t_{reward}$$

$$= R_{1}{TAC}_{ref}\left( t \right)+\left( k_{2}-\frac{R_{1}k_{2}}{\left( 1+{BP}_{ND_{Reward}} \right)} \right)Q(t) if t\geq t_{reward}$$

Where

- $t$=0 represents the start of the scan at time of bolus injection
- $h_{neutral}\left( t \right)$ and $h_{reward}\left( t \right)$ are the exponential system impulse responses in the reward and neutral states, respectively;
- $Q\left( t \right)$ is a time-dependent discrete convolution of the reference TAC with the system response kernels accounting for the reward and neutral conditions;
- $TAC\left( t \right)$ represents the TAC of a given atlas region;
- ${TAC}_{ref}\left( t \right)$ represents the TAC for the cerebellar reference region (measured from the cerebellar regions of the AAL3 atlas, excluding regions labeled as vermis);
- $R_{1}$ is an estimated parameter of the SRTM model representing the ratio of kinetic transport rates from plasma to free-tracer tissue compartments in the TAC region studied and reference region;
- $k_{2}$ is an estimated parameter of the SRTM model representing the kinetic transport rate from free-tracer tissue to plasma compartments in the TAC region studied;
- ${BP}_{ND_{Neutral}}$and ${BP}_{ND_{Reward}}$ are estimated parameters representing the non-displaceable binding potential in each of the two task states, Neutral and Reward, respectively;
- $t_{reward}$ is the time at which the reward task is begun (usually 42 minutes into the study but varied for individual subjects based on the recorded task start time; the baseline binding potential is estimated from injection up to start of reward block).

For each TAC, the two-part model was fitted with a custom MATLAB script applying a nonlinear least-squares fit to the SRTM model. Thus, for each subject and for each hypothesized atlas region, we obtained estimates of BP_ND_Neutral_ and BP_ND_Reward_.

For regional estimates from the six-region striatal atlas, the cerebellum reference TAC from the AAL3 atlas was used as the reference TAC.

F. BP_ND_ Voxel Maps

To perform voxel-wise analysis in the common MNI space, a set of BP_ND_ maps was created for each subject. For each subject, the 70 motion-corrected PET images were smoothed with an 8mm Gaussian kernel to reduce noise for individual voxel fits. A mask was applied to screen out voxels with little PET activity. Then, a TAC was obtained for each voxel over the 70 time frames, and the two-part SRTM model defined in part E was applied to obtain BP_ND_Neutral_ and BP_ND_Reward_ for each voxel within the brain mask. This resulted in subject-space maps of BP_ND_Neutral_ and BP_ND_Reward_. A map of fit quality in each voxel, based on the coefficient of determination from each fit, was also produced and checked as a quality-control procedure. There were no issues observed with fit quality in the striatal regions.

The subject-space maps were then transformed to the MNI space using the transformations of part C. This allowed the individual subject maps to be compiled in the common MNI space, creating maps of mean and variance of BP_ND_R-N_ (Reward-Neutral) for each group (Control and ASD) and corresponding z-score maps.

**Supplemental Materials III.** Sub-region analysis of left caudate nucleus / left putamen PET cluster

Visual inspection of the 87-voxel left caudate nucleus / left putamen PET BP_ND_ cluster that differentiated groups revealed that the cluster also contained voxels in white matter. To address whether the BP_ND_ values derived from this cluster were strongly influenced by voxels in white matter , we sub-divided the cluster into two smaller clusters that were anatomically constrained on the basis of whether voxels were in the left caudate nucleus or the left putamen based on the Harvard-Oxford subcortical probabilistic atlas. Visual inspection revealed that thresholding these probabilistic regions at 7% produced masks that maintained as many voxels as possible in the masks without assigning the same voxel to both regions. The result was a 32-voxel region in the left caudate nucleus and a 26-voxel region in the left putamen (see Table). These regions were queried by examining baseline BP_ND_ (i.e., during the neutral task block) and BP_ND_ during the reward task. The graphs in the Figure indicate that both sub-regions recapitulated the pattern observed in the larger combined region.

BP_ND_ values during the neutral and reward task blocks for the ASD and control groups for the left caudate nucleus and left putamen sub-regions derived from the combined left caudate nucleus / left putamen PET cluster reported in the main text. For both subregions, the Group $\times$ Condition interaction effect is significant (left caudate nucleus: interaction p<0.01; left putamen interaction p<0.006).

| Size, magnitude, and location of the larger left caudate nucleus/putamen cluster, as well as anatomically constrained left caudate nucleus and left putamen subregion. |
| --- |

| **Cluster Label** | **Cluster Size (voxels)** | **Z Max** | **X** | **Y** | **Z** |
| --- | --- | --- | --- | --- | --- |
| Left caudate nucleus / putamen | 87 | 3.95 | -18 | 2 | 10 |
| Anatomically constrained left caudate nucleus | 32 | 3.28 | -16 | 4 | 12 |
| Anatomically constrained left putamen | 26 | 3.54 | -20 | 2 | 10 |

**Supplemental Materials IV.** PET region-of-interest analysis

Region-of-interest analysis of striatal regions from the 1mm striatum structural atlas ^6^ are presented in the **Figure**. Regions were transformed to each subject’s PET space and resampled to the 344$\times$344$\times$127 PET grid and mapped using linear interpolation in order to weight edge voxels for partial volume effects.


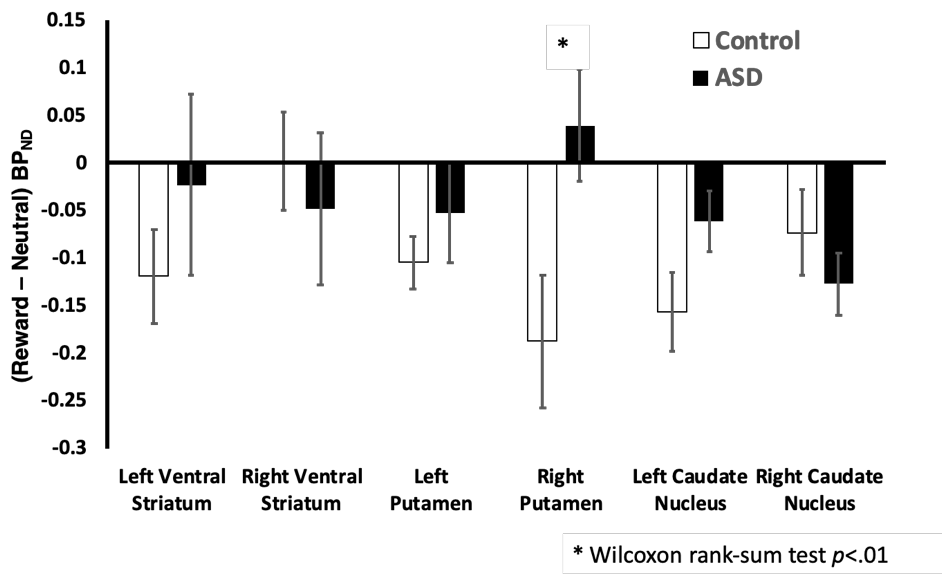


Region-of-interest analysis of striatal regions from the 1mm striatum structural atlas ^6^. Values represent relative change in BP_ND_ between the neutral and reward conditions.

**Supplemental Materials V.** Power and effects size analyses

We calculated the statistical power and effect sizes for each of the central PET findings representing Group (Control, ASD) X Condition (Reward, Neutral) differences in BP_ND_ values in three striatal clusters (left putamen, right putamen, and left caudate/putamen, see Figure 2). To calculate the statistical power of these Group $\times$ Condition interaction effects, these interaction effects were first analyzed via a linear mixed model in the R system for statistical computing (Ver. 3.5.2; R Development Core Team, 2009), where participant was treated as a random effect and Condition and Group were treated as fixed effects. In these three clusters**,** the interaction effects are significant as shown in the table below. The estimator of the interaction terms, which follows a t-distribution with 20 degrees of freedom, was used to compute statistical power (i.e., 1-the probability of a Type II error). Setting the probability of Type I error to α=0.05, from the effect sizes (i.e., the |t-values|), the power of each test was determined (see the table below). For all three clusters, the effect size is at least 2.90 and power is at least 0.79.

|  | Estimate | S.E. | DF | \|t-value\| (effect size) | p-value | power |
| --- | --- | --- | --- | --- | --- | --- |
| Left Putamen | -0.19 | 0.065 | 20 | 2.90 | 0.0089 | 0.79 |
| Right Putamen | -0.23 | 0.078 | 20 | 2.94 | 0.0081 | 0.80 |
| Left Caudate/Putamen | -0.17 | 0.048 | 20 | 3.60 | 0.0018 | 0.93 |

**Supplemental Materials VI.** Exploratory fMRI activation results

Whole brain activation ASD<control results. **Left:** During reward anticipation, the ASD group showed decreased activation in a cluster in the left putamen. **Right**: During reward outcomes, the ASD group showed decreased activation in several cortical regions, including the anterior cingulate gyrus (indicated by the arrow in the figure).

Z

Z

| Functional activation clusters for whole brain ASD<control voxel-wise analyses. There were no clusters that demonstrated ASD>Control differences for either contrast | | | | | | | |  |
| --- | --- | --- | --- | --- | --- | --- | --- | --- |
| **Contrast** | **Region** | **Cluster Size (voxels)** | **Brodmann Area** | **X** | **Y** | **Z** | **Z Max** | |
| *Anticipation* | Left Postcentral Gyrus | 306 | 2 | -48 | -28 | 38 | 2.99 | |
|  | Left Cerebellum (V) | 122 |  | -10 | -54 | -24 | 3.19 | |
|  | Right Middle Frontal Gyrus | 55 |  | 44 | 6 | 62 | 2.99 | |
|  | Right Parahippocampal Gyrus | 48 | 35 | 24 | -8 | -32 | 3.15 | |
|  | Left Postcentral Gyrus | 46 |  | -66 | -16 | 40 | 2.96 | |
|  | Right Cerebellum (I-IV) | 44 |  | 12 | -42 | -30 | 2.82 | |
|  | Right Precentral Gyrus | 41 |  | 24 | -12 | 50 | 2.82 | |
|  | Left Putamen | 34 |  | -28 | 4 | 14 | 3.13 | |
|  | Right Supramarginal Gyrus | 29 |  | 58 | -24 | 50 | 3.04 | |
|  | Right Middle Frontal Gyrus | 28 | 47 | 56 | 44 | -18 | 2.76 | |
|  | Right Precentral Gyrus | 27 |  | 36 | -8 | 56 | 2.91 | |
|  | Posterior Cingulate Gyrus | 26 | 31 | 0 | -26 | 48 | 2.82 | |
| *Outcome* | Right Occipital Pole | 4469 |  | -6 | -98 | 10 | 6.13 | |
|  | Anterior Cingulate Gyrus | 1486 |  | -4 | 18 | 48 | 4.22 | |
|  | Right Middle Frontal Gyrus | 1163 | 6 | 69 | -2 | 48 | 4.48 | |
|  | Left Cerebellum (Crus II) | 879 |  | -32 | -74 | -52 | 4.74 | |
|  | Precentral Gyrus | 671 |  | -2 | -34 | 70 | 4.23 | |
|  | Left Precentral Gyrus | 558 |  | -44 | 0 | 52 | 4.89 | |
|  | Right Cerebellum (Crus I) | 460 |  | 28 | -64 | -28 | 3.9 | |
|  | Right Occipital Pole | 4469 |  | -6 | -98 | 10 | 6.13 | |

**Supplemental Materials VII.** Exploratory generalized psychophysiological interactions (gPPI) results

| Regions showing greater connectivity in the ASD relative to control group during reward anticipation with the PET-derived left putamen seed region. | | | | | | |  |
| --- | --- | --- | --- | --- | --- | --- | --- |
| **Region** | **Cluster Size (voxels)** | **X** | **Y** | **Z** | **Cluster-level**  **family-wise error-corrected p-value** | | |
| Left Frontal Orbital Cortex | 57 | -32 | 30 | -18 | .0272 | | |
|  |  |  |  |  |  |  |  |

Voxel-wise whole-brain gPPI analyses revealed a significant ASD>Control group difference in connectivity between the PET-derived left putamen seed region and a target region in the left orbital frontal cortex during reward anticipation. There were no group differences in connectivity with any other PET-derived striatal seed regions during reward anticipation or during reward outcomes.

**Supplemental Materials VIII.** Task reaction time and valence ratings

A Group (ASD, Control) $\times$ Cue (neutral, small reward, medium reward, large reward) ANOVA on reaction times revealed no main effect of Group or Group $\times$ Cue interaction, *p*’s > 0.64, but a main effect of Cue, F(3,20)=3.28, *p*<0.03. Follow-up t-tests examining reaction time differences between cue conditions, collapsing across groups, revealed that reaction times were slower to the neutral cue than the other conditions, *p*’s<.01, but there were no reaction time differences between the small, medium, and large reward cues.

A Group (ASD, Control) $\times$ Cue (neutral, small reward, medium reward, large reward) ANOVA on valence ratings revealed no main effect of Group or Group x Cue interaction, *p*’s > 0.10, but a main effect of Cue, F(3,60)=8.23, *p*<0.0021. Follow-up t-tests examining valence rating differences between cue conditions, collapsing across groups, revealed that valence ratings were higher for the large reward cue than the medium and small reward cues, and higher for the medium reward cue than small reward cue, and higher for the large reward cue than the neutral cue, *p*’s<.05.

A Group (ASD, Control) $\times$ Outcome (no reward, small reward, medium reward, large reward) ANOVA on valence ratings revealed no main effect of Group or Group $\times$ Outcome interaction, *p*’s > 0.38, but a main effect of Outcome, F(3,60)=82.51, *p*<0.0001. Follow-up t-tests examining valence rating differences between outcomes, collapsing across groups, revealed that valence ratings differed between all four outcomes, *p*’s<.0001.

The **Figure** below illustrates task reaction times to cues and valence ratings elicited by cues and rewards.

**
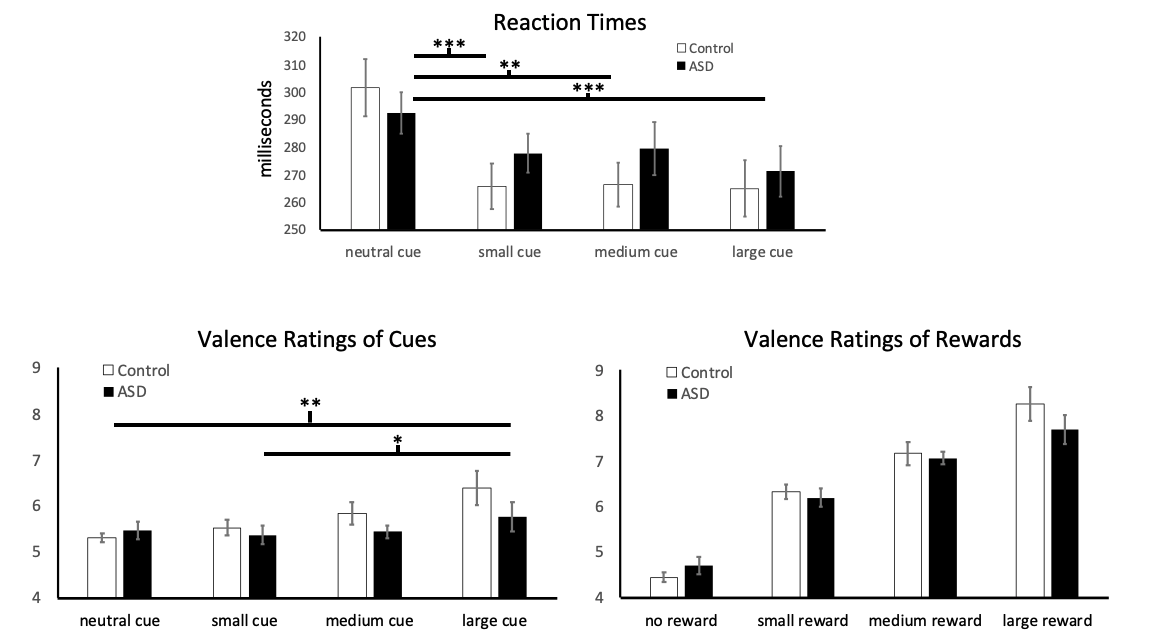
**

Task reaction times to cues and valence ratings elicited by cues and rewards. Valence ratings were made using a 9-point Likert scale with anchors of “very negative” (1) and “very positive” (9) at the ends and “neutral” (5) in the center. Note: the ‘neutral cue’ refers to the cue that predicted the no reward outcome, whereas the ‘small’ ‘medium’, and ‘large’ cues predicted the fifty cent, one dollar, and five-dollar outcomes, respectively. Conditions differed from each other at *: p<.05; **: p<.01; ***: p<.005. Error bars represent standard errors of the mean.

**Supplemental Materials IX.** Correlations between striatal dopamine binding and ASD symptom severity

Exploratory correlational analyses (Pearson’s *r*) in the ASD group considered associations between (reward>neutral) BP_ND_ values in the four striatal PET clusters that differentiated groups, and ADOS-2 calibrated severity scores^54^, SRS total t-scores, and RMITE scores. Two associations in the hypothesized direction emerged: decreased phasic DA release to incentives in the left and right putamen were related to worse performance on the RMITE, a measure of theory-of-mind, in the ASD group (*p*=0.03 and *p*=0.04, respectively). These results were only significant at an uncorrected significance threshold.

Exploratory correlational analyses (Pearson’s *r*) in the ASD group between the strength of functional connectivity values and clinical measures were not significant.


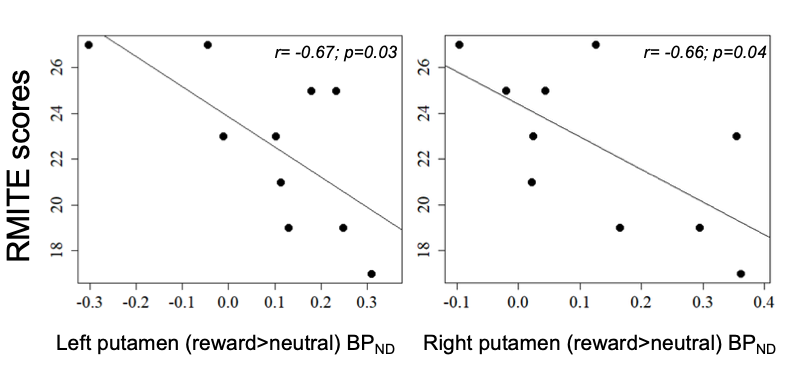


Association between the left and right putamen (reward>neutral) BP_ND_ values and the Reading the Mind in the Eyes” Test, Revised Version (RMITE) scores in the ASD group. Higher RMITE scores indicate better performance. Positive BP_ND_ values indicate decreased phasic dopamine release to incentives. Pearson’s *r* correlations, *p*<0.05, uncorrected for multiple comparisons.

**References**

1. Izquierdo-Garcia D, Hansen AE, Forster S, Benoit D, Schachoff S, Furst S *et al.* An SPM8-based approach for attenuation correction combining segmentation and nonrigid template formation: application to simultaneous PET/MR brain imaging. *J Nucl Med* 2014; **55**(11)**:** 1825-1830.

2. Ladefoged CN, Law I, Anazodo U, St Lawrence K, Izquierdo-Garcia D, Catana C *et al.* A multi-centre evaluation of eleven clinically feasible brain PET/MRI attenuation correction techniques using a large cohort of patients. *Neuroimage* 2017; **147:** 346-359.

3. Frackowiak RSJ. *Human brain function*. Academic Press: San Diego, 1997, xiii, 528 p.pp.

4. Fedorov A, Beichel R, Kalpathy-Cramer J, Finet J, Fillion-Robin JC, Pujol S *et al.* 3D Slicer as an image computing platform for the Quantitative Imaging Network. *Magn Reson Imaging* 2012; **30**(9)**:** 1323-1341.

5. Ashburner J. A fast diffeomorphic image registration algorithm. *Neuroimage* 2007; **38**(1)**:** 95-113.

6. Tziortzi AC, Searle GE, Tzimopoulou S, Salinas C, Beaver JD, Jenkinson M *et al.* Imaging dopamine receptors in humans with [11C]-(+)-PHNO: dissection of D3 signal and anatomy. *Neuroimage* 2011; **54**(1)**:** 264-277.

7. Buchert R, Thiele F. The simplified reference tissue model for SPECT/PET brain receptor studies. Interpretation of its parameters. *Nuklearmedizin* 2008; **47**(4)**:** 167-174.
